# The Citation Merit of Scientific Publications

**DOI:** 10.1371/journal.pone.0049156

**Published:** 2012-11-13

**Authors:** Juan A. Crespo, Ignacio Ortuño-Ortín, Javier Ruiz-Castillo

**Affiliations:** 1 Departamento de Economía Cuantitativa, Universidad Autónoma de Madrid, Madrid, Spain; 2 Departamento de Economía, Universidad Carlos III de Madrid, Madrid, Spain; Aalto University, Finland

## Abstract

We propose a new method to assess the merit of any set of scientific papers in a given field based on the citations they receive. Given a field and a citation impact indicator, such as the mean citation or the 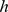-index, the merit of a given set of 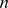 articles is identified with the probability that a randomly drawn set of 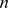 articles from a given pool of articles in that field has a lower citation impact according to the indicator in question. The method allows for comparisons between sets of articles of different sizes and fields. Using a dataset acquired from Thomson Scientific that contains the articles published in the periodical literature in the period 1998–2007, we show that the novel approach yields rankings of research units different from those obtained by a direct application of the mean citation or the 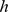-index.

## Introduction

The scientific performance of a research unit (a university department, research institute, laboratory, region, or country) is often identified with its publications and the citations they receive. There are a variety of citations-based specific indices for assessing the impact of a set of articles. Among the most prominent are the mean citation and the 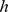-index, but there are many other possibilities. Regardless of the citation impact indicator used, the difficulty of comparing units that produce a different number of papers -even within a well-defined homogenous field- must be recognized. To better visualize the problem consider a concrete example. Suppose that we use we use as indicator the mean citation. Consider the articles published in Mathematics in 1998 and the citations they receive until 2007. The mean citation of papers published in Germany and Slovenia are 5.5 and 6.4, respectively. However, Germany produced 1,718 articles and Slovenia only 62. According to the mean citation criterion the set of Slovenian articles has greater relative impact than the German set. We will see, however, that according to the novel proposal introduced in this paper the performance exhibited by Germany has greater merit than that of Slovenia. No doubt this is an extreme example, but highlights a general difficulty that is present when comparing research units producing a different number of papers in the same field. Furthermore, we show that this difficulty in comparing sets of different sizes persists even if they are large. Thus, the problem in our example is not due to the small number of papers published in Slovenia. This difficulty is even more apparent for citation impact indicators that are size dependent, such as the 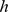-index [1], [2].

Comparisons across fields are even more problematic. Because of large differences in publication and citation practices, the numbers of citations received by articles in any two fields are not directly comparable. Of course, this is the problem originally addressed by relative indicators recommended by many authors [3]–[11]. A convenient relative impact indicator is the ratio between the unit’s observed mean citation and the mean citation for the field as a whole. Thus, after normalization, mean citations of research units in heterogeneous fields become comparable [12]. However, we argue that, as in the previous example of Germany and Slovenia, comparisons using normalized mean citations do not capture the citation merit of different set of articles.

The main aim of this paper is to propose a method to measure the citation merit of a set of articles a research unit publishes in a homogeneous field over a certain period. It should be clarified at the outset that the merit is conditional on the indicator used (mean, 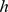-index, median, percentage of highly cited papers, etc.) and on the set of articles used as reference, which we will call “population of interest” (usually all the world articles published in a field in a given period). Thus, a given set of papers in a certain field and time period may have different merit depending on the citation impact indicator used. Given a citation impact indicator, our method allows for comparisons between sets of papers of different sizes and fields. Thus, we will be able to make statements like “The scientific publications of Department X in field A have a greater citation merit than the publications of Department Y in field B”.

Our method is based on a very simple and intuitive idea. Given a field and a citation impact indicator, the merit of a given set of 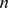 articles is identified with the percentile in which its observed citation impact lies on the distribution of citation impact values corresponding to all possible subsets of 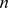 articles in that field. Suppose, for example, that the impact indicator is the mean citation, and that the population of interest is equal to all articles published in the world in a certain period in that field. In this case, the merit of a given set of 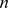 papers is given by the probability that a randomly drawn set of 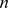 articles in that field has a lower mean citation. Note that, since the merit of a set of papers of a research unit is associated with a percentile (or a probability), it is possible to compare two such percentiles for research units of different sizes working in different fields.

This method resembles that used in other areas such as, for example, Pediatrics where the growth status of a child is given by the percentile in which his/her weight lies within the weight distribution for children of the same age. In our case “same age” is equivalent to “same number of articles”. There is, however, an essential difference: in our case we do not compare the performance of a given research unit with 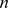 articles with the performance of other existing research units with a similar number of articles, but with the distribution generated by all possible subsets of 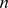 articles from a given pool of articles.

A related idea that also distinguishes between citation impact and citation merit can be found in [13] for the evaluation of scientific excellence in geographical regions or cities. The citation impact indicator they use is the percentage of articles in a city that belong to the top-10% most-highly cited papers in the world. As they say “the number of highly-cited papers for a city should be assessed statistically given the number of publications in total”. Thus, the scientific excellence of a city depends on the comparison between its observed and its expected number of highly cited papers.

The 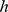-index has become very popular because it can be seen as capturing both quantity and quality. The original proposal by Hirsch [14] was designed for the evaluation of individual researchers, but it can be easily extended to research units (A research unit has 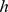-index 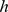 if 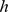 of its articles have at least 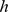 citations each, and the remaining articles have no more than 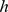 citations each). However, due to its dependence on the number of articles, research units that have more articles also tend to have higher 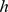-index values. For the different institutions they study, Molinari and Molinari [1] show that an universal relation emerges across institutions that enable them to empirically decompose the 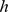-index as a product of a size independent factor 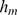 and a size dependent one 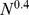, where 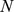 is the number of papers. This factor 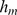 is then used among others by Kinney [2] who compares the scientific output of several U.S. institutions. In our case, we do not need to rely on any empirical estimation of the relation between 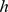-index and the size 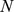 (moreover our methodology can be applied to all citation impact indicators).

In order to implement our method, a large dataset with information about world citation distributions in different homogeneous fields is required. In most of this paper, we use a dataset acquired from Thomson Scientific, consisting of all articles published in 1998–2007, and the citations they received during this period. We show that our approach yields rankings of research units quite different from those obtained by a direct application of the mean citation and the 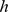-index.

## Methods

Consider a homogeneous scientific field (for example, Nuclear Physics, Molecular Biology, etc.). Suppose that we want to compare the relative merit of two sets of articles 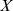 and 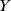. Denote by 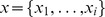 the vector of citations received over a fixed period by the 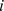 articles in 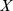, and by 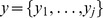 the corresponding vector of citations for the 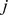 articles in 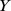. Denote by 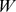 the set of articles used as a “population of interest”, and by 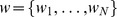 the vector of citations of the 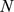 articles in 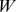. We require that 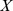, 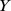 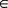 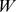. In most applications in the paper we take 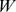 as the set of all articles published in the world in a given year in that field.

We next need some citation impact indicator 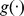 such as, for example, the mean citation or the 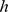-index. The mean citation is perhaps the most often-used indicator, but recently the 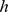-index has also become popular. Our method is silent about which is the most appropriate citation impact indicator. Given an indicator, we could compare 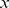 and 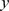
’s impact by comparing the numbers 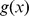 and 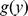. As indicated in the Introduction, such a direct comparison has important drawbacks and is often misleading. Thus, we propose a way to compare the merit of any two vectors of citations using the information 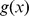, 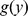, 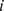, 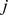, and 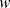.

Denote by 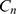 the set of all subsets of 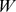 of size 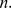 We take 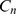 as our sample space and the corresponding 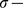
algebra is given by all the subsets of 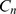, i.e. 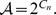 We establish a probability function 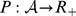 satisfying that all the simple events are equiprobable, i.e.
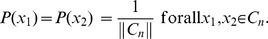
where 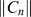 denotes the (finite) number of elements in the set 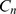, i.e 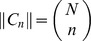.

Given the measure space 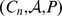 we define the random variable 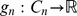 which is just our chosen impact indicator 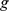 restricted to sets of 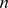 articles. The cumulative distribution function (CDF) of 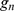, 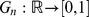, is defined by.
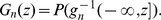



Note that 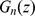 denotes the probability that a subset of 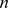 articles from 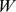 has a vector of citations 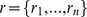 such that 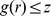.

### 

#### Definition

*The citation merit of a set of* 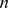 *papers* 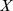 *with citation vector* 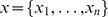 *is given by* 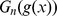. *We write* 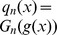.

Thus, we associate the citation merit of 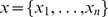 with the percentile in which the number 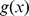 lies in the distribution 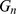.

It should be emphasized that to determine the merit of a set of articles we just have to calculate a percentile (or probability) which does not require any statistical inference exercise.

In many cases we know the analytical expression of the function 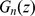. For instance, in the case of the 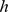-index, the function 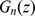 can be calculated exactly as described by Molinari and Molinari ([1], Equations A3, A6). This is a combinatorial formula that only requires to know the vector of citations in the population of interest, 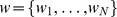.

However, in other instances, it might be difficult to calculate the analytical expression of 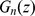. In these cases, one could approximate 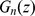 by taking 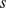 random draws of 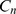. As in many empirical applications the cardinality of 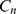 is large, the number of draws should be large (in our applications we use 100,000). Thus whenever it is difficult to compute the combinatorial formula that gives the exact value of 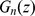 we proceed as follows: Let 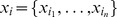, 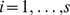, be the vector of citations obtained in the 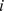-th draw. Apply the impact indicator to each of these 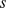 sets and denote by 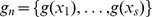 the resulting vector. Let 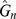 be the distribution function associated to such vector, so that 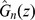 gives the percentage of components in vector 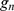 with a value equal or less than 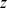. Given a database with the information of 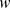, this is a feasible and simple approach to approximate the probability 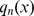.

To further motivate our citation merit definition, think of the following hypothetical example. Consider a given field and period and suppose that each article has only one author, and each author has written only one article. Suppose that the research unit is a university department that has published 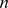 papers, obtaining a citation impact level equal to 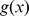. Suppose that instead of the actual department composition the chair could hire 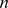 persons from the pool of world researchers who have written a paper in the same field, and let 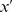 be the corresponding vector of citations. Assume that the chair of the department hires these 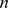 people in a random way (so there is no difference from what a monkey would do). What would the probability be that 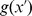, the citation impact level associated with such hypothetical random hiring, is lower than the actual value 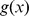
? Such probability is our citation merit value 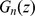.

Coming back to the example presented in the Introduction about the articles in Mathematics of Slovenia and Germany and judging by their mean citation of 6.3 and 5.5, Slovenia ranks higher than Germany. However, the merit values we obtain for the sets of papers of these two countries are 85.30 and 97.00, respectively. The probability that a set of 62 papers, randomly chosen from the pool of all papers published in Mathematics, have a mean lower than 6.3 is 85.30%, whereas the probability that a set of 1,718 papers have a mean lower than 5.5 is 97.00%. Thus, although the mean citation for Slovenia is higher than the mean citation for Germany, its merit is lower.

It is important to note that the result in this example is not just due to the fact that the “sample size” for Slovenia is very small (62 papers). Our empirical results provide many similar type of examples for larger number of papers. Consider, for example, the field of Engineering. Taiwan has 1,882 articles and mean citation of 5.58. Scotland has 610 articles and basically the same mean citation, 5.54. However, the merit of these two sets of articles are 31.20 and 36.00, respectively. Thus, even in cases with “large” number of articles our merit function might rank sets of articles differently from the rank obtained by the mean citation.

Given a citation impact indicator and a population of interest, the method just introduced allows us to compare sets of articles in the same field, and rank all of them in a unique way. Moreover, since the merit definition is associated with a percentile in a certain distribution, we can also make meaningful merit comparisons of sets of articles from different fields.

## Results

We use a dataset acquired from Thomson Scientific, consisting of all publications in the periodical literature appearing in 1998–2007, and the citations they received during this period. Since we wish to address a homogeneous population, in this paper only research articles are studied. After disregarding review articles, notes, and articles with missing information about Web of Science category or scientific field, we are left with 8,470,666 articles. For each article, the dataset contains information about the number of citations received from the year of publication until 2007 (see [15], for a more detailed description of this database).

We only consider two citation impact indicators: the mean citation, and the 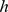-index. As already indicated, in the case of the 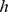-index, our merit function 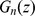 can be calculated analytically. In the case of the mean, the precise combinatorial formula for 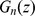 is complicated and, in practice, it is not feasible given the large size of our datasets. Then we use the approximation 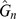 described above.

Since the mean and the standard deviation of 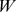 are known, one could think of approximating 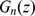 using the Central Limit Theorem, at least for research units with large numbers of articles. However, for all scientific fields the distribution of 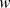 is heavily skewed [15]–[19], and the underlying distribution might not have a finite variance, so that the Central Limit Theorem could fail even for research units with a large number of articles. (We have indeed checked that for the scientific fields used in the paper, and the sample sizes given the number of papers published by the research units considered, the distribution of the means of random samples is far from a normal distribution).

### Countries

**Figure 1:**
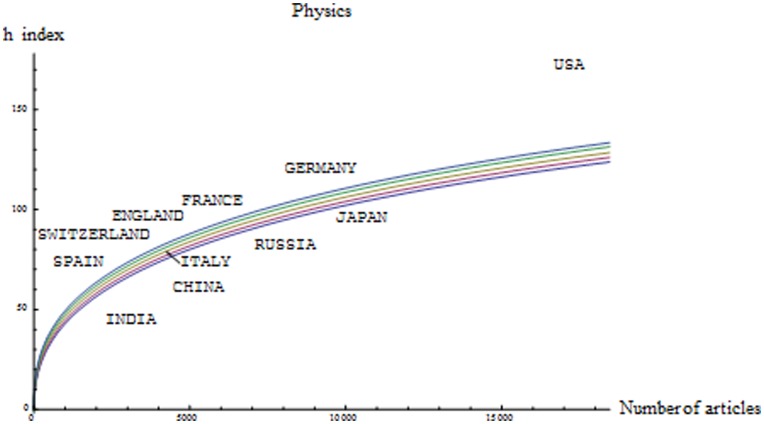
Field of Physics. Papers published in 1998. Curves represent for each number of articles the corresponding value of the 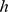-index for which the merit is 10.00, 25.00, 50.00, 75.00 and 90.00 respectively. Points represent the number of papers and the values of the 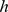-index for different countries.

In a first exercise, research units are countries, and the homogeneous fields are identified with the 22 broad fields distinguished by Thomson Scientific, 20 in the natural sciences, and two in the social sciences. In an international context we must confront the problem raised by cooperation between countries: what should be done with articles written by authors belonging to two or more countries? Although this old issue admits different solutions (see [20] for a discussion), in this paper we side with many other authors in following a multiplicative strategy (see [21]–[23]). Thus, in every internationally co-authored article a whole count is credited to each contributing area. Excluding the Multidisciplinary field, for each of the remaining 21 fields we compute the citation merit of the papers of each country according to the mean citation and the 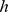-index, taking as the population of interest all papers published in the world in the corresponding field. We exclude the Multidisciplinary field because of the high heterogeneity of some of its journals. In doing so we exclude many high-impact articles published in, for example, Nature, Science and PNAS. One should incorporate such articles to their corresponding fields. This is, however, a laborious task that is beyond the scope of this paper. Figure 1 illustrates an example of our methodology when citation impact is measured by the 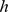-index for the articles published in 1998 in the field of Physics, their citations until 2007, and a selection of countries. For each different value of 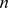, Figure 1 shows the value of the 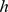-index corresponding to percentiles 10, 25, 50, 75 and 90 of the corresponding distribution 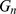, as well as the number of articles published by each country and its associated 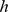-index.

Note that by just observing the 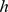-index of, for example, Japan, France, Germany, and Switzerland, it is difficult to assess their relative merit. The reason, of course, is that the 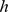-index is highly dependent on the number of articles. Thus, since Japan (9,600 articles), France (6,056), and Germany (9,598) produce more articles than Switzerland (2,028), they also have a higher 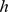-index. However, with our method we are able to compare the publications of these countries using 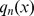, the percentile where the observed 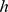-index lies. It turns out that obtaining by chance an 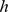-index as high as the one of Switzerland -with 2,028 papers- is a much more “unlikely” event than obtaining the 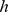-index of any of the other three countries with their corresponding number of articles. Thus, our method assigns more merit to Switzerland (basically percentile 100) than to Japan (percentile 5.40), France (percentile 99.00), and Germany (percentile 99.90). Figure 1 also shows that the U.S. produces the largest number of articles, has the highest 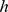-index and, according to our methodology, basically reaches the 100 percentile. This is a feature that appears in most of the 21 fields that we have analyzed.

Table 1 continues with the case of articles published in Physics in 1998 and equivalent tables for the remaining 20 fields are available as supplementary information to this paper, Tables S1. For the forty countries with the largest production, the tables provide the 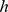-index, the mean citation, the corresponding 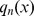 values, and the confidence intervals for our approximation in the case of the mean citation. For example, Italy has an 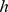-index of 81, the sixth highest value in our sample. But if we look at the merit index 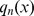, it falls to the eleventh position. It is observed that any of the two impact indices and its corresponding merit index 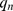 produce different rankings. There are many examples where the discrepancy between the two is very large. Thus, our methodology delivers outcomes that are quite different from those obtained by the direct use of the mean citation or the 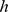-index criterion.

**Table 1:** Field of Physics.

		*h-index*		Mean citation	
Country	Articles	*h*-index	Percentile	Mean	Percentile
	(*n*)		(*q_n_*)	citation	(*q_n_*)
USA	18267	173	100.00	19.52	100.00
					(100.00, 100.00)
JAPAN	9600	99	5.40	11.96	36.71
					(36.411, 37.008)
GERMANY	9598	114	99.90	15.54	100.00
					(100.00, 100.00)
RUSSIA	8116	75	0.00	7.49	0.00
					(0.000, 0.000)
FRANCE	6056	97	99.00	15.10	100.00
					(100.00, 100.00)
ENGLAND	4890	90	99.30	14.68	100.00
					(100.00, 100.00)
CHINA	4294	59	0.00	7.80	0.00
					(0.000, 0.000)
ITALY	4086	81	87.70	14.40	99.99
					(99.987, 99.998)
INDIA	2239	45	0.00	8.40	0.00
					(0.000, 0.000)
SPAIN	2089	67	98.40	15.15	99.94
					(99.929, 99.958)
SWITZERLAND	2028	81	100.00	21.96	100.00
					(100.00, 100.00)
SOUTH KOREA	1911	51	0.00	9.77	0.00
					(0.000, 0.000)
CANADA	1895	61	76.40	14.98	99.86
					(99.841, 99.887)
POLAND	1794	52	0.90	12.12	56.33
					(56.027, 56.641)
NETHERLANDS	1504	61	99.30	14.60	99.24
					(99.187, 99.294)
BRAZIL	1481	46	0.00	9.42	0.00
					(0.000, 0.000)
AUSTRALIA	1373	49	6.80	11.69	35.23
					(34.934, 35.530)
ISRAEL	1330	56	94.90	15.19	99.62
					(99.581, 99.657)
SWEDEN	1250	52	68.10	15.72	99.82
					(99.793, 99.846)
UKRAINE	1250	29	0.00	4.97	0.00
					(0.000, 0.000)
TAIWAN	1160	39	0.00	7.74	0.00
					(0.000, 0.000)
BELGIUM	933	48	82.80	14.01	94.72
					(94.585, 94.863)
AUSTRIA	751	47	98.00	16.00	99.27
					(99.218, 99.324)
DENMARK	746	55	100.00	19.58	99.99
					(99.981, 99.994)
MEXICO	692	29	0.00	7.65	0.00
					(0.000, 0.000)

Papers published in 1998 and their citations until 2007.

For each country the merit index 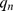 is obtained using as 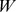 the total set of papers published in the World in Physics in 1998 (45718 papers) and their citations received until 2007. For the 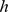-index the value 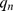 is computed analytically and for the mean citation 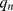 is approximated using the simulation approach described in the paper with 100,000 random subsets. The corresponding 95% binomial confidence intervals appear in brackets. The figures have been rounded up to three decimal places.

In some cases our methodology cannot discriminate enough between countries with very high merit indices. Consider for example the case of Clinical Medicine in Table 2, where Column 4 shows the merit index for a selection of countries when the citation impact is measured by the 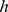-index. All these countries, except Germany, have a very similar merit index close to 100%. The reason for this result is that we are using as a population of interest all articles published in the world, and the quality of the articles published by this selection of countries is much higher than that of the rest of the world. Therefore, there are not many corresponding subsets of articles with citation impact as high as those observed in the countries in question. One possible way to discriminate among these “very high quality” sets of articles is to take as population of interest, 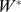, only articles published in these countries. Column 5 in Table 2 shows the citation merit index in this case. Notice that when W contains all the papers published in the world France reaches the 99.4% percentile. However, in the case of W* –a set of papers of a much higher quality than the W set– basically about half of all subsets of size 13,822 have an 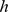-index higher than the one of France (140). Thus, in this case France’s percentile is 55.3%. Notice that changing the population of interest might produce a re-ranking of the citation merit. When 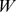 is used, England obtains a higher citation merit than Belgium. However, the opposite is the case when the population of interest is 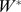. This possibility of re-ranking is not surprising since our notion of merit is based on the comparison of the observed 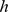-index with the probability of obtaining sets of articles with lower h-indices. Such probability depends on the distribution function associated to the population of interest.

**Table 2:** Field of Clinical Medicine.

			*W* = 155178	W* = 119390
(1)	(2)	(3)	(4)	(5)
Country	Articles	*h*-index	Percentile	Percentile
	*n*		(*q_n_*)	(*q_n_*)
USA	56463	284	100.00	100.00
GERMANY	13822	144	16.68	0.00
ENGLAND	13243	162	99.99	92.03
FRANCE	9556	140	99.45	55.40
ITALY	7471	140	100.00	99.89
CANADA	6297	143	100.00	100.00
NETHERLANDS	4789	123	100.00	99.96
AUSTRALIA	4081	107	99.53	71.14
SWEDEN	4030	114	100.00	99.56
SWITZERLAND	3080	105	100.00	99.80
BELGIUM	2470	94	99.98	96.82
SCOTLAND	2016	90	100.00	99.47
FINLAND	1946	92	100.00	99.97
DENMARK	1841	86	99.99	98.43
NORWAY	1187	71	99.76	90.80

Papers published in 1998 and their citations until 2007.

Column (4): the merit index 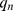 is obtained using as 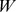 the total set of papers published in the World in Clinical Medicine in 1998 (155.178 papers) and their citations received until 2007. Column (5): the merit index 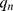 is obtained using as 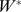 the total set of papers published in these 15 countries in Clinical Medicine in 1998 (119.390 papers) and their citations received until 2007. The figures have been rounded up to three decimal places.

### University Departments and Laboratories

It could be argued that the broad fields so far analyzed are, in effect, too heterogeneous, a fact that may well diminish the value of our results. In this subsection we present comparisons of the merit of the publications of some selected university departments and laboratories in two more homogeneous scientific sub-fields (Thomson Scientific assigns articles to 219 Web of Science categories through the journals where they have been published, but many journals are simultaneously assigned to two or more categories. For a discussion of the strategies to deal with the problem raised by the multiple assignments of articles to Web of Science categories, see [24]). Tables 3 and 4 show the performance of some institutions in the sub-fields of Neuroscience and Economics, respectively (the data on the papers published by members of these departments has been obtained from the Web of Science of Thomson Scientific). The tables show the number of papers, the 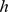-index, the mean citation, and the corresponding 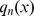.

**Table 3:** Sub-field of Neurosciences.

		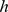 -index		Mean citation	
(1)	(2)	(3)	(4)	(5)	(6)
Institution	Articles	*h*-index	Percentile	Mean	Percentile
	(*n*)		*(q_n_*)	citation	(*q_n_*)
Yale	209	53	100.00	48.01	100.00
					(100.00, 100.00)
Massachusetts Gen Hosp	186	62	100.00	69.26	100.00
					(100.00, 100.00)
Howard Hughes Med Inst	172	76	100.00	90.58	100.00
					(100.00, 100.00)
Stanford University	133	43	100.00	55.29	100.00
					(100.00, 100.00)
Rockefeller University	73	35	100.00	49.32	100.00
					(100.00, 100.00)
MIT	64	31	99.99	57.05	100.00
					(100.00, 100.00)
Salk Inst Biol Studies	59	34	100.00	78.34	100.00
					(100.00, 100.00)
Brigham & Womens Hosp	44	23	99.77	39.09	98.13
					(98.044, 98.212)
National Insitute of Aging (NIA)	40	19	89.50	32.95	89.82
					(89.637, 90.011)
Amgen	29	19	99.89	62.28	99.96
					(99.946, 99.971)
Smithkline Beecham Pharmaceut	24	17	99.86	52.75	99.49
					(99.442, 99.531)
Rush Presbytherian St Lukes Med	24	14	86.79	32.33	84.05
					(83.821, 84.275)
University Fribourg	20	13	92.49	46.55	98.06
					(97.971, 98.141)
Princeton	20	14	98.32	43.05	96.64
					(96.523, 96.747))
Beth Israel Med Ctr	19	12	84.81	40.74	94.94
					(94.804, 95.076)
Natl Inst Med Res	18	12	90.67	46.94	97.66
					(97.569, 97.757)
Mayo Clin Jacksonville	16	14	99.98	43.56	95.56
					(95.431, 95.687)
Max Delbruck Ctr Mol Med	16	13	99.69	34.38	85.51
					(85.296, 85.732)
Cold Spring Harbor Lab	12	10	98.14	56.33	98.44
					(98.366, 98.520)

Papers published in 1998 and their citations until 2007.

For each institution the merit index 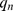 is obtained taking 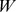 as the total set of papers published in the World in Neuroscience in 1998 (21876 papers) and their citations received until 2007. For the 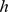-index the value 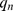 is computed analytically and for the mean citation each 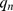 is approximated using the simulation approach described in the paper with 100,000 random subsets. The corresponding 95% binomial confidence intervals appear in brackets. The figures have been rounded up to three decimal places.

**Table 4:** Sub-field of Economics.

		*h*-index		Mean citation	
(1)	(2)	(3)	(4)	(5)	(6)
Institution	Articles	*h*-index	Percentile	Mean	Percentile
	(*n*)		(*q_n_*)	citation	(*q_n_*)
Chicago Univ	77	27	100.00	45.90	100.00
					(100.00, 100.00)
Berkeley Univ	70	17	99.82	20.27	99.82
					(99.792, 99.844)
Penn Univ	67	23	100.00	21.70	99.89
					(99.871, 99.912)
Northwestern Univ	65	17	99.92	17.37	99.15
					(99.090, 99.204)
MIT	62	31	100.00	38.73	100.00
					(100.00, 100.00)
Univ Maryland	62	15	98.71	14.21	97.72
					(97.628, 97.813)
Stanford	57	21	100.00	24.12	99.92
					(99.899, 99.953)
Univ Minnesota	42	14	99.87	13.40	94.43
					(94.287, 94.571)
Princeton	36	18	100.00	34.61	99.99
					(99.9777, 99.992)
Duke Univ	28	12	99.94	16.46	97.07
					(96.963, 97.172)
Univ Virginia	26	9	92.45	17.85	98.00
					(97.909, 98.083)
Univ Carlos III Madrid	26	7	47.40	5.84	21.39
					(21.130, 21.639)
Boston Univ	22	13	99.99	34.27	99.69
					(99.651, 99.721)
Univ Iowa	22	8	89.53	9.95	72.47
					(72.197, 72.751)
Boston Col	20	10	99.86	9.10	65.69
					(65.400, 65.988)
Univ Oklahoma	18	6	61.28	5.17	19.08
					(18.833, 19.321)
Univ Pompeu Fabra	17	8	98.11	11.88	83.07
					(82.836, 83.300)
Univ Texas Austin	15	9	99.94	17.87	95.71
					(95.584, 95.836)
Insead	14	8	99.65	24.79	98.82
					(98.753, 98.887)
Univ Miami	10	5	87.45	11.50	79.95
					(79.7000, 80.196)

Papers published in 1998 and their citations until 2007.

For each institution the merit index 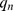 is obtained taking 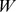 as the total set of papers published in the World in Economics in 1998 (7542 papers) and their citations received until 2007. For the 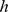-index the value 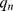 is computed analytically and for the mean citation each 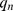 is approximated using the simulation approach described in the paper with 100,000 random subsets. The corresponding 95% binomial confidence intervals appear in brackets. The figures have been rounded up to three decimal places.

As before, there are significant discrepancies between the ranking according to the direct citation impact indicator (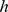-index or mean citation) and our merit function 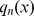. Notice that many departments get a value of 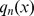 equal or very close to 100%. As already explained in the case of Clinical Medicine in Table 2, this is not surprising since all of them are top departments and the probability that we obtain by chance articles with such a high mean citation, or 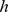-index, from the set of world papers must be close to zero. As before, this lack of discrimination among top departments can be fixed by considering a different population of interest 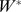. In addition, for the case of the mean citation we can increase the number of random subsets used to estimate 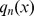. So far, in our empirical results we have always drawn 100,000 random subsets (for each 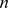). This might be more than enough for intermediate percentiles but not for percentiles very close to 100. However, given the purpose of this paper, we find of no practical importance that, for example, the differences in Table 3 between percentiles 99.96 and 99.49 are not statistically significant.

## Discussion

In this paper we have proposed a new simple and intuitive method to assess the citation merit of any set of scientific papers in any field. One advantage of our approach is that it can be applied to a variety of problems. For example, it might be applied to rank scientific journals. The merit of a given journal that publishes 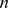 articles in a year in a given field would be given by the probability that a subset of 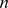 articles in that field are of lower quality according to some criterion as the mean citation or the 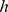-index (note that the merit of a journal is not the same as the merit of the authors who publish in the journal). A second advantage is the possibility of comparisons of the scientific merit of research units in different fields. This can be done because the merit of each research unit is associated with a probability (or percentile) that might be reasonable to compare across different fields.

As far as the international cooperation is concerned, it is well known that domestic and international publications are characterized by very different citation rates. Therefore, using whole counts as we have done in this paper, or following [20] recommendation in favor of using fractionalized counts to calculate citation indicators at the national level, might make a significant difference that it would be convenient to investigate.

In the empirical application of the method we have used two well-known and vastly different citation impact indicators: the mean citation and the 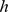-index. However, recall that, given their high skewness, the upper and lower parts of citation distributions are typically very different. Consequently, average-based indicators -such as the mean citation- may not adequately summarize these distributions. On the other hand, both the 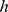-index and many of the indicators of the same family have been criticized by some authors (see [25]–[27]). Therefore, it may be worthwhile to study the merit of research units according to some of the new indicators that are rapidly being suggested (see [26], [28]–[32]).

It is important to note that our approach is not trying to make any inference on the underlying model explaining the scientific output of the different units. For an overall assessment of the relative merit or performance of a research unit we should take into account many other variables, such as the budget, number of researchers, etc. Two research units with the same merit according to a set of citation indicators as understood in this paper may vastly differ in the productivity of its research staff or, more generally, in the efficiency with which scientific results are obtained from a complex input vector. Thus, we only provide a method to assess a research unit’s performance in a certain dimension, quite independently of the underlying model explaining why different units produce scientific publications of different citation impact and citation merit.

## Supporting Information

Tables S1 **Analysis of the 40 countries with the largest number of papers in the corresponding field.**
(XLSX)
